# Simulation of Virtual Redundant Sensor Models for Safety-Related Applications

**DOI:** 10.3390/s22030778

**Published:** 2022-01-20

**Authors:** Peter Peniak, Karol Rástočný, Alžbeta Kanáliková, Emília Bubeníková

**Affiliations:** Department of Control and Information Systems, Faculty of Electrical Engineering and Information Technology, University of Žilina, 01026 Žilina, Slovakia; karol.rastocny@feit.uniza.sk (K.R.); alzbeta.kanalikova@feit.uniza.sk (A.K.); emilia.bubenikova@feit.uniza.sk (E.B.)

**Keywords:** virtual redundant sensor, fail-safe, safety-related application, fusion function, digital twin, MQTT, simulation, software tool RabbitMQ, safety, availability

## Abstract

Applications of safety-related control systems demand reliable and credible inputs from physical sensors, therefore there is a need to extend their capabilities to provide a validated input with high availability. Our main idea is to insert virtual sensors between physical sensors and the control system’s logic. The created solution can validate the values of real sensors and with the use of multiple virtual sensors we can achieve high availability in addition, therefore our solution is entitled as a virtual redundant sensor. It works by the digital twin’s concept and uses fusion function to calculate validated results. The fusion function is used to transform the measured values from the physical sensors according to designed numerical models. The selection of a numerical model with assigned fusion functions can be performed via the WEB-based graphical user interface. Proposal of the numerical model is created and validated on the experimental workplace with emulation of physical sensors and MQTT integration (smart IoT sensors). The results of testing have shown that our solution can be applied to validate the values of physical sensors. Proposed fusion functions calculated results according to the selected model in all cases, while non-standard cases were handled according to our definition. In addition, the high availability concept with a group of two virtual sensors has proven fast recovery and availability of results for safety-related applications as well.

## 1. Introduction

There are processes (e.g., in industry and transport) that are associated with the occurrence of a dangerous (hazardous) event that can result in damage to human health and the environment, or significant material damage. Such undesirable events or their consequences may be prevented by the application of technical and organizational security measures. Technical safety measures also include safety-related control systems (SRCSs). Sensors are also a part of such systems.

The safety-related applications in process control require credible and validated inputs from physical sensors (PSs) to control safety-related processes using methods that guarantee not only the required level of safety level but also high availability. Therefore, our article will deal with virtual redundant sensors. 

Virtual sensor (VS) is, by one of the available definitions, a digital twin of the physical device, or logical/digital representation of a real physical sensor, whose capabilities are enriched. Although the virtual sensor is seen as a real sensor, it exists just logically or is provided by the IT infrastructure (Cloud/Edge). The virtual sensor can even exist when the physical sensor is not present. It is a similar approach as virtualization of computers (virtual machines), networks (VLANs), and software components. 

**Figure 1:**
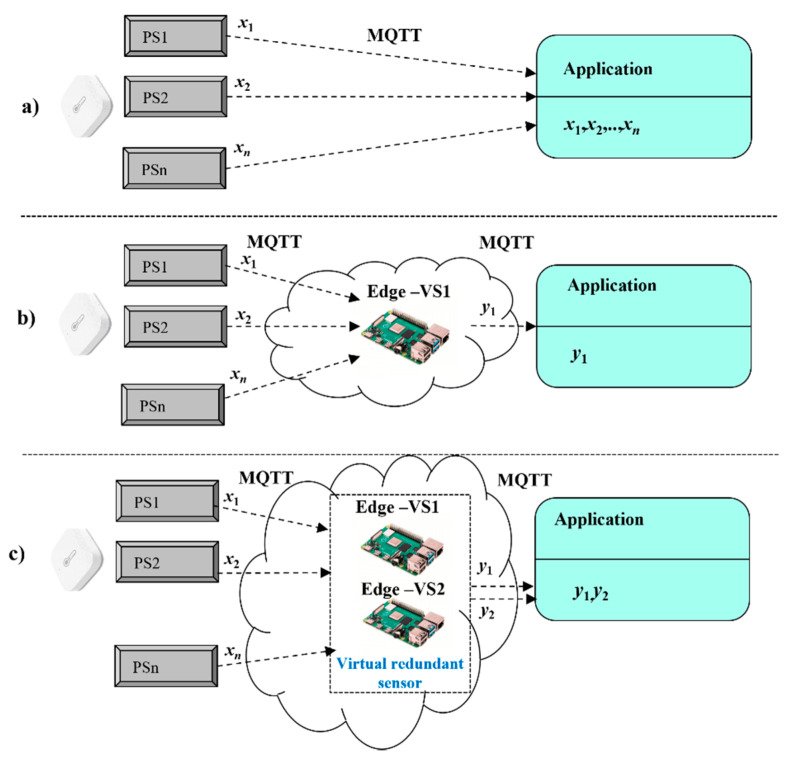
Different models for safety-related applications and sensors (**a**) classical model, (**b**) original model and (**c**) extended model.

The virtual redundant sensor is our new term defined by us in [1] because we want to suggest the use of the virtual sensors not only as a representant of the physical sensors with validation of physical sensors’ values but also to achieve their high availability by applying for a redundancy. The classical approach assumes that each application includes the necessary features to support the validation of sensors values, as shown by Figure 1a. This approach naturally leads to the higher complexity of applications and requires repetition of the same programming steps with each application. The solution according to Figure 1a assumes redundancy only at the level of physical sensors. 

This article extends our initial work [1], where we developed the original concept for virtual redundant sensor and created its basic numerical model and validated solution based on a dedicated Edge device with an MQTT broker. In order to simplify the design of safety-related applications, we introduced the dedicated Edge device for processing all sensors’ values with the calculation of validated results, based on a selected numerical model (*m*oo*n*), as shown by Figure 1b. The solution according to Figure 1b contains redundancy not only at the level of physical sensors but also software redundancy applied inside the virtual sensor. 

Although we have been able to validate physical sensor values, the created solution has been vulnerable in case of Edge failure. In such a case, the application would not be able to work with sensors’ values and would be inoperable. Therefore, there was a need to revise our original concept and elaborate on the target solution. First, our essential idea is to extend the original concept of the virtual redundant sensor with flexible calculation models according to the required use cases, without the necessity to create the new solutions, case by case. Second, fail-safe conditions require a solution with high availability, so we are proposing the new model, shown in Figure 1c. It is based on the group of two dedicated Edge devices, which are mirrored via AMQP protocol, and could take-over services each other to support applications in fail-safe approach. The solution according to Figure 1c contains redundancy not only at the level of physical sensors but also at the level of virtual sensors.

## 2. Virtual Sensors

The concept of the Internet of Things and the Industrial Internet of Things addresses connectivity and communication anytime, anywhere, for anything and anyone [2]. On the one hand, it facilitates data acquisition, on the other hand, failures in these systems can cause accidents and undermine credibility in the eyes of the public. Therefore, it is necessary to focus on the means of solving reliability such as fault avoidance, fault tolerance, fault rectification [3,4].

The physical sensors can measure values with errors, and this can lead to the instability of other systems that need data from the measuring sensors and can lead to an accident. Today, we already know systems for detection, and fault isolation diagnostics (FID). The aim of FID systems is to reach a system that is resistant to failures (failure, erroneous measurements), which will retain the functionality of the existing system or systems that receive data from that system. Such a system control approach is called the fault-tolerant control (FTC) approach [5,6]. Sensors are increasingly found in a wide range of applications, such as intelligent mobile devices, traffic management, automotive systems, industry [7,8,9], healthcare, environmental monitoring, health monitoring, and the like.

**Figure 2:**
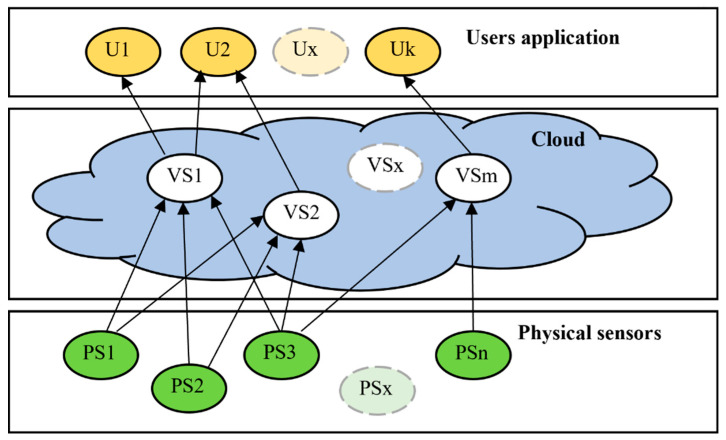
Virtual sensors connection.

The virtual sensor is a software emulation of the physical sensor which retrieves values from basic physical sensors. Virtual sensors work often in a Cloud system (Software as a Service) and can work in multiple configurations in conjunction with physical sensors: one PS connected to one VS (one-to-one), one PS connected to multiple VSs (one-to-many), multiple PSs connected to one VS (many-to-one), multiple PSs connected to multiple VSs (many-to-many) [10], in Figure 2.

The development of virtual sensors has significantly helped in the control processes of industrial, transport, and other processes, especially when seen from the point of view of accuracy and availability. In the literature review, virtual sensors are defined as [11]:

- Mathematical models for predicting process behavior;
- Mathematical and statistical models to enrich the physical sensors values;
- A combination of new analytics methods to generate new value in real-time.

The same review [11] of the literature and the studies lists the criteria that were used in the implementation of virtual sensors:

- In controlled process monitoring-fault detection, detail fault, estimation, performance degradation;
- In process optimization–optimization parameters, scheduling, supply chain.

In [12,13,14,15,16,17], it is possible to find some interesting applications of virtual sensors in areas such as:

- Traffic—vehicle suspension control by means of virtual sensors based on a neural network to determine the estimate vehicle unsprang mass relative velocity [12];
- Environment—virtual sensing for real-time monitoring of surface and groundwater sources for irrigation purposes [13];
- Health—use of a virtual sensor for safety monitoring of a person’s position as a replacement for a real position sensor, provided that the position matches within the active radius of the sensor [14];
- Working environment—temperature monitoring in the indoor environment (offices, living spaces, industrial environment) and prediction of temperature development using genetic programming [15];
- Industry—A virtual sensor for detecting and resolving a human physical collision with conventional industrial robots using two torque observers [16];
- Agriculture—implementation of virtual sensors designed to monitor the temperature in greenhouses [17].

In [18,19,20], it is possible to find applications for the use of virtual sensors to increase the functional safety or diagnosis of virtual sensor failures, such as:

- Maintaining the functional safety state of direct current converters (DC-DC) converters using hardware-physical sensors and techniques based on advanced extended Kalman filter (EKF) and virtual sensors [18];
- Fault detection and diagnosis sensor of the chiller in air conditioning systems using virtual sensors constructed by a long-short-term memory network [19];
- Fault detection and diagnostics in ventilation units using virtual sensors designed to measure deviations of values [20] and many others.

Based on the above information, it is appropriate to implement virtual sensor applications in terms of:

- Optimization, prediction, or data analysis;
- Safety, availability, and fault detection.

## 3. The Numerical Model of the Virtual Sensor for Safety-Related Applications

**Figure 3:**
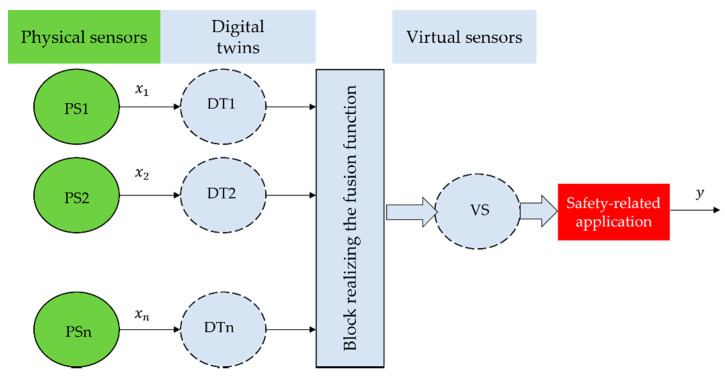
Virtual sensor model for safety-related application.

According to the proposed concept, the virtual sensor model is based on digital twins. Physical sensors are assigned to their digital twins and provide the actual values that are measured by the sensors. The fusion function (1) is used to transform the measured values (values from the physical sensors) to the value provided by the virtual redundant sensor. The value thus obtained from the virtual sensor is then provided to safety-related applications, as shown in Figure 3.

In general, the function of fusing signals from sensors can be described (1), where y is the resulting signal from the virtual redundant sensor, $x_{i}$ is the signal from the *i*-th physical sensor, and *n* is the number of the physical sensors.

(1) $$y=F\left(x_{1},\;x_{2},\;\ldots ,\;x_{n}\right).$$

**Figure 4:**
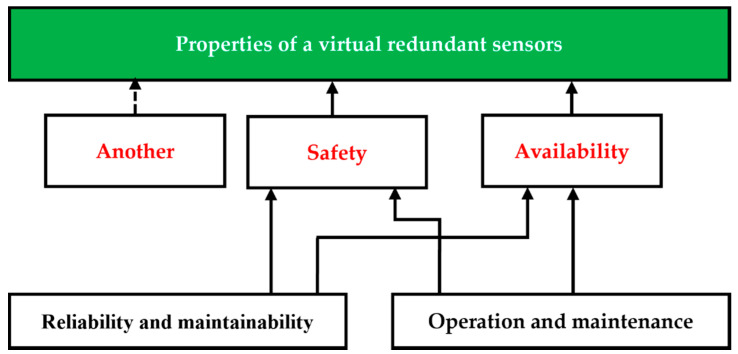
The relationship between the RAMS parameters and virtual redundant sensors.

The aim of SRCSs (which in a broader sense that can include the concept of a virtual redundant sensor) is to have the required availability and safety features. These properties are expressed by the so-called RAMS (Reliability, Availability, Maintainability, Safety) parameters. The relationship between these parameters is shown in Figure 4. The concept of a virtual redundant sensor does not preclude its use in applications where high availability and minimum safety requirements are required.

The amount of hardware and software redundancy depends on requirements for the availability and the safety integrity of such a virtual redundant sensor. In this article, we consider the processing of values in the virtual redundant sensor from a maximum of three physical sensors. The prerequisite for using the virtual redundant sensor is that the signals from the physical sensors correspond to the same physical quantity.

Depending on the application in which the virtual redundant sensor is to be used, signal processing from *n* physical sensors can be realized in order to:

- Increase the safety of values at the output of the virtual redundant sensor (mathematical model *n* out of *n*, in the following text *n*oo*n*, where n>1);
- Increase the safety and availability of values at the output of the virtual redundant sensor (mathematical model *m* out of *n*, in the following text *m*oo*n*, where 1<m≤n).

Physical sensor redundancy can only be used to increase the safety of information from physical sensors (model *n*oo*n*, if n=2, then model is 2oo2). Then the function (1) has the form:

(2) $$y_{2\mathrm{oo}2}\left(t\right)=F_{2\mathrm{oo}2}\left(x_{1}\left(t\right),\;x_{2}\left(t\right)\right).$$

The requirement of safety demands the definition of:

- Tolerable range (*TR*) of difference of values from physical sensors;
- A method of selecting a signal value at the output of the virtual redundant sensor if the physical sensors provide signals of different values; in general, depending on the application, either the smallest value or the largest value can be considered safer; in the case of a logic signal, it is usually a signal with a log. level 0 [21,22,23]; in the next part of the article, we assumed that the safest value is considered to be the smallest value (3) of the physical sensor signals (a similar consideration can be made if the safest value would be considered the largest value of the physical sensor signals);
- The safe state of the virtual redundant sensor (output signal value) if the signal values from the physical sensors are out of the tolerable range; in this case, the virtual redundant sensor will leave this safe state only as a result of corrective intervention by an authorized worker.

Example of using the min{} function: Let a dangerous state be considered to be a state when the liquid level in the tank is below a defined critical value. The liquid level is measured by two physical sensors. A safe response (opening of the liquid filling valve) is activated when at least one of the physical sensors reports that a critical value has been reached.

Example of using the max{} function: Let a dangerous state be considered to be a state when the liquid level in the tank is above a defined critical value. The liquid level is measured by two physical sensors. The safe response (opening of the liquid filling valve) is activated when at least one of the physical sensors reports that a critical value has been reached.

Then

(3) $$if\;\left|x_{1}\left(t\right)-x_{2}\left(t\right)\right|\leq TR,\;\mathrm{then}\;y_{2\mathrm{oo}2}\left(t\right)=min\left\{x_{1}\left(t\right),\;x_{2}\left(t\right)\right\},\;if\;\left|x_{1}\left(t\right)-x_{2}\left(t\right)\right|>TR,\;\mathrm{then}\;y_{2\mathrm{oo}2}=0.$$

If it is a logic signal, the resulting value from the virtual redundant sensor can be determined based on the relationship:

(4) $$y_{2\mathrm{oo}2}=x_{1}\cdot x_{2}.$$

If the disagreement of the logic signal levels at the output of the physical sensors ($x_{1}\neq x_{2}$) lasts longer than the defined tolerable disagreement time $t_{TD}$, then $y_{2\mathrm{oo}2}=0$ and this value can change only due to the corrective intervention of the authorized worker.

Theoretically, it is also possible to consider the *n*oo*n* model, where n>2, but such a solution comes into consideration only in a very specific situation.

If the redundancy of physical sensors is used in order to increase the safety and availability of values from physical sensors (it is about model *m*oo*n*, if m=2 and n=3), then it is the model 2oo3. Then the function (1) has the form:

(5) $$y_{2\mathrm{oo}3}\left(t\right)=F_{2\mathrm{oo}3}\left(x_{1}\left(t\right),\;x_{2}\left(t\right),\;x_{3}\left(t\right)\right).$$

If it is an analog signal and it applies:

$$\left|x_{1}\left(t\right)-x_{2}\left(t\right)\right|=k_{1}\leq TR,$$

(6) $$\left|x_{1}\left(t\right)-x_{3}\left(t\right)\right|=k_{2}\leq TR,$$

$$\left|x_{2}\left(t\right)-x_{3}\left(t\right)\right|=k_{3}\leq TR,$$

then

(7) $$y_{2\mathrm{oo}3}\left(t\right)=min\left\{x_{1}\left(t\right),\;x_{2}\left(t\right),\;x_{3}\left(t\right)\right\}.$$

If that is true

(8) $$\left(k_{1}\leq TR\right)\wedge \left(k_{2}>TR\right)\wedge \left(k_{3}>TR\right),$$

then

(9) $$y_{2\mathrm{oo}3}\left(t\right)=min\left\{x_{1}\left(t\right),x_{2}\left(t\right)\right\}$$

and the physical sensor PS3 (Figure 3) is logically isolated from the virtual redundant sensor ($x_{3}\left(t\right)=0$) and the virtual redundant sensor operates in the degraded mode (model 2oo2) until taking the corrective intervention of the authorized worker. Subsequently, based on this intervention, the virtual redundant sensor goes into full mode (model 2oo3).

If that is true

(10) $$\left(k_{1}>TR\right)\wedge \left(k_{2}\leq TR\right)\wedge \left(k_{3}>TR\right),$$

then

(11) $$y_{2\mathrm{oo}3}\left(t\right)=min\left\{x_{1}\left(t\right),x_{3}\left(t\right)\right\}$$

and the physical sensor PS2 (Figure 3) is logically isolated from the virtual redundant sensor ($x_{2}\left(t\right)=0$) and the virtual redundant sensor operates in the degraded mode (model 2oo2) until taking the corrective intervention of the authorized worker as a result. Subsequently, based on this intervention, the virtual redundant sensor goes into the full mode (model 2oo3).

If that is true

(12) $$\left(k_{1}>TR\right)\wedge \left(k_{2}>TR\right)\wedge \left(k_{3}\leq TR\right),$$

then

(13) $$y_{2\mathrm{oo}3}\left(t\right)=min\left\{x_{2}\left(t\right),\;x_{3}\left(t\right)\right\}$$

and the physical sensor PS1 (Figure 3) is logically isolated from the virtual redundant sensor ($x_{1}\left(t\right)=0$) and the virtual redundant sensor operates in the degraded mode (model 2oo2) until taking the corrective intervention of the authorized worker. Subsequently, based on this intervention, the virtual redundant sensor goes into full mode (model 2oo3).

In all other combinations of signal values from physical sensors, the output $y_{2oo3}\left(t\right)=0$ until corrective intervention is taken by an authorized worker. In the case of a logic signal, the resulting value from the virtual redundant sensor can be determined by voting based on the relationship:

(14) $$y_{2\mathrm{oo}3}=x_{1}\cdot x_{2\;}+x_{1}\cdot x_{3}+x_{2}\cdot x_{3}.$$

If the disagreement of the logic signal levels at the output of the physical sensors ($x_{1}\neq x_{2}$, or $x_{1}\neq x_{3}$, or $x_{2}\neq x_{3}$) lasts longer than the defined tolerable disagreement time $t_{TD}$ and $y_{2\mathrm{oo}2}=0$, the same procedure is followed as in the case of analog signal if a non-functional physical sensor can be identified, it is disconnected and the virtual redundant sensor goes from the full mode (model 2oo3) to the degraded mode (model 2oo2).

Theoretically, it is also possible to consider the *m*oo*n* model, where m>2 and n>3, but such a solution comes into consideration only in a very specific situation.

Calculations and processing of values must take place in an environment characterized by a high safety level that corresponds to the safety level of the signal provided by the virtual redundant sensor.

In Section 3 of this article, we provide only a basic mathematical description of the problem. The algorithms actually used are significantly more complex due to the minimization of hazardous (undesirable) states in collision situations due to the change of signals at the outputs of physical sensors.

## 4. Extended Model of the Virtual Redundant Sensor

As explained in Section 3, the proposed model of the virtual sensor is primarily designed to provide the validation of physical sensors’ values with the selectable fusion function and mathematical model according to Formulas (1) till (14). Yet, there is a risk of virtual sensor failure, which would lead to the overall malfunction of the connected application. Our virtual sensor had to be extended with respect to redundancy, which would ensure its availability. There are several options how to achieve the requested redundancy. This article deals with two possible approaches on how to implement redundancy. Our extended model consists of two identical virtual sensors that are implemented on dedicated independent physical Edge devices, performing validation of physical sensors’ values by using the same fusion Function (1). If one of them fails, the other must step in and support the application with sensors’ values. 

In this article, the focus was paid on two alternative approaches:

1. Virtual redundant sensors with mirroring;
2. Virtual redundant sensors with duplexing.

**Figure 5:**
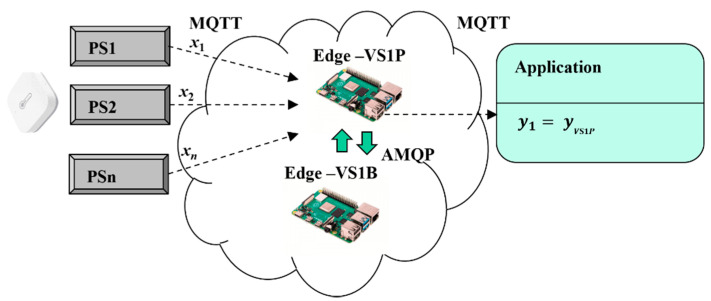
Virtual redundant sensor model with mirroring.

Virtual redundant sensors with mirroring was our first approach (Figure 5). Let us assume that one of the virtual sensors is always active VS1P (virtual primary sensor), while the second one VS1B (virtual backup sensor) is a backup. VS1B just mirrors all physical sensors’ values from VS1P via replication process. AMQP connection is used for replication of sensors values $(x_{1},\;x_{2},\;\ldots ,\;x_{n}),$ which are mirrored to associated AMQP queues. VS1B can hold values even if the connection with VS1P is broken. VS1P works in active mode and communicates with physical sensors and applications via MQTT and performs validation of sensors values according to the chosen model (*m*oo*n*). The validated results are provided by VS1P (15). If VS1P fails, VS1B will step in to support application with sensors’ values.

(15) $$y_{1}=y_{VS1P}.$$

**Figure 6:**
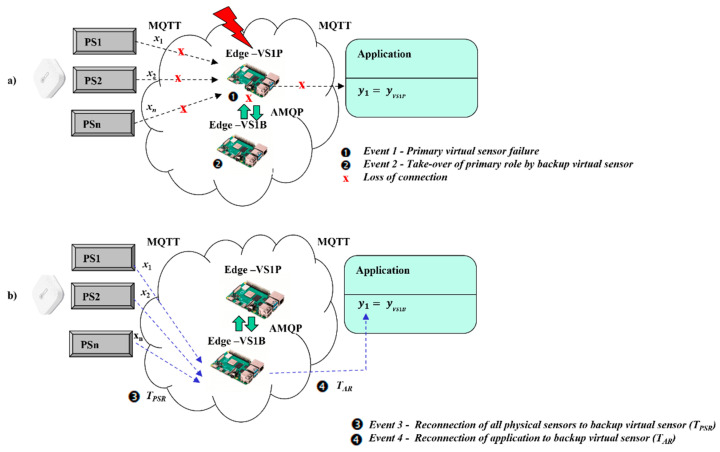
Virtual redundant sensor model with mirroring in case of VS1P failure.

Figure 6a illustrates the operations of the virtual redundant sensor in case of VS1P failure. After VS1P is broken (Event 1) all communications channels are closed. VS1B recognizes the unavailability of VS1P and takes over the role of the active virtual sensor including his IP address. Temporarily unavailable virtual sensor VS1P will be switched by common configuration to the backup role in case of a restart to prevent looping of connections (Event 2). After reconnection of application (Event 3) and physical sensors (Event 4), the validation process (*m*oo*n*) can be restarted again based on mirrored physical sensors values in AMQP queues, as shown by Figure 6b.

(16) $$y_{1}=y_{VS1B}.$$

Although the virtual redundant sensor model with mirroring enables fast recovery according to fail-safe principles, there is a still need for some transition time $T_{T}$ to recover operation after time when VS1P had failed. All the physical sensors and applications have to be configured with a forced MQTT reconnection process to MQTT broker in case the connection is lost. As soon as a failure of VS1P is recognized by VS1B, a recovery process is initiated. VS1B takes-over IP address of VS1P and the forced reconnection process starts by physical sensors and applications. The overall reconnection time of physical sensors $T_{PSR}$ can be expressed by (17), as a maximum of reconnection times of all physical sensors $T_{PS1R},\;T_{PS2R},\;\ldots ,\;T_{PSnR}$. Then overall transition time $T_{T}$ can be calculated as maximum from reconnection time of application $T_{AR}$ and reconnection time of physical sensors $T_{PSR}$ according to (18). After transition time is reached, the virtual redundant sensor is recovered and results are provided by VS1B. Formula (19) covers safety-related requirement of applications.

(17) $$T_{PSR}=\max\{T_{PS1R};\;T_{PS2R};\;\ldots ;\;T_{PSnR}\},$$

(18) $$T_{T}=\max\{T_{PSR};\;T_{AR}\},$$

(19) $$T_{T}\leq T_{ST},$$

where $T_{ST}$ is the tolerable time interval during which application can work without validated virtual sensors’ values (according to an application specific safety-related requirements).

To overcome the mentioned imperfections of the mirroring approach, an alternative virtual sensor model with duplexing was suggested. The main goal is to mitigate the temporary unavailability of virtual redundant sensors and overall transition time ($T_{T}=0$) so that safety-related requirements of application could be kept. There are again two virtual sensors used (VS11, VS12), but in contrast to the previous model, they are both active modes having connections with physical sensors and applications. It means that the validation of physical sensor values ($x_{1},\;x_{2},\;\ldots ,\;x_{n}$) is performed simultaneously with the use of selected fusion functions (*m*oo*n*) by two virtual sensors on Edge devices. There are several aspects to be taken into consideration:

- An application has to be able to receive two validation results ($y_{VS11}$, $y_{VS12}$);
- A possible delay with the delivery of the results has to be handled properly ($T_{VS11},\;T_{VS12}$);
- A pairing of the results has to be insured by the setting of the result identificator ($R_{id}$);
- The virtual sensors have to communicate together and align the results with parameter (p); if p=0 both virtual sensors can provide results, if p=1 the result is valid only from one virtual sensor.

**Figure 7:**
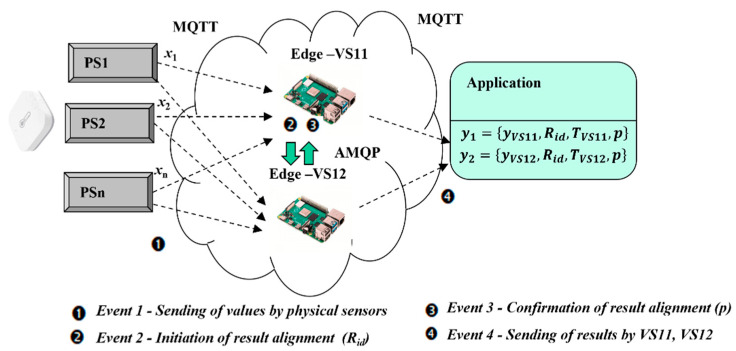
Virtual redundant sensor model with duplexing approach.

The principle of the duplexing approach is explained in Figure 7. Physical sensor values are received by both sensors (Event 1). Alignment of the results requires the arbiter function, which is performed by the leading sensor. Arbiter function is pre-selected on VS11, but it can be taken-over by VS12 in case of VS11 failure. As soon as the leading virtual sensor calculates the result, the incremental identifier $R_{id}$ is assigned and sent with input parameters to VS12 (Event 2). VS12 assigns for the result the same $R_{id}$ and confirms it to VS11 via AMQP (Event 3). VS11 lets result parameter without change (p=0), as validated by both sensors. In case of communication with VS12 is not possible, VS11 sets p=1 indicating that results from VS12 to be ignored (possible malfunction). Both virtual sensors can send their results together (Event 4) with $R_{id}$, p in slightly different times based on network conditions ($T_{VS11},\;T_{VS12}$), according to Formulas (20) and (21). In the case of parameter p=0, the application can use any of the results. In the case of parameter p=1, application has to use only associated result from the same virtual sensor.

(20) $$y_{1}=\left\{y_{VS11},\;R_{id},\;T_{VS11},p\right\},$$

(21) $$y_{2}=\left\{y_{VS12},\;R_{id},\;T_{VS12},\;p\right\}.$$

## 5. Experimental Workplace for Simulation of Virtual Redundant Sensors

The proposed numerical model of the virtual redundant sensor, described in Section 4, underwent experimental validation in our laboratory. First, we designed experimental solution to test the various numerical models of virtual redundant sensors having taken into consideration the possible implementation scenarios. The following aspects have been considered during the design phase of our experimental solution:

- Physical sensors have to support the most common IoT protocols;
- Integration layer for sensors has to be broadly supported and known;
- Virtual redundant sensor shall support various implementation scenarios;
- Mathematical models shall be configurable and with the modular approach.

Based on these preconditions, we have chosen MQTT as an integration layer for physical sensors, acting as IoT devices. MQTT works on top of the TCP/IP (Transmission Control Protocol/Internet Protocol) protocol stack as a lightweight broker-based publish/subscribe messaging protocol. MQTT protocol is based on “Publish/Subscribe” model with central “Broker instance”, which creates the concept of a “global data space” that is accessible to all interested applications [24,25,26]. All communication is represented as reads and writes to the global data space. This chapter has aligned all symbols between software screens and text so that it can be better understood. As well, we use symbol VS as a simplified name for virtual redundant sensors in our next text.

The physical sensors are connected to MQTT broker as a publisher, writing measured values (*x_i_*) into associated Topics_*x_i_*. Virtual redundant sensor, on the contrary, is connected as subscriber, reading all values from Topics_*x_i_*. Afterwards, the values of physical sensors can be evaluated according to the selected numerical model (YM00N) and transformed according to associated formulas ($F_{m\mathrm{oo}n}$) to the new value of virtual redundant sensor *VS_i_* (*y*_1_), and the virtual redundant sensor can support multiple calculations in the same time (for example *y*_1_ means 1st calculation).

**Figure 8:**
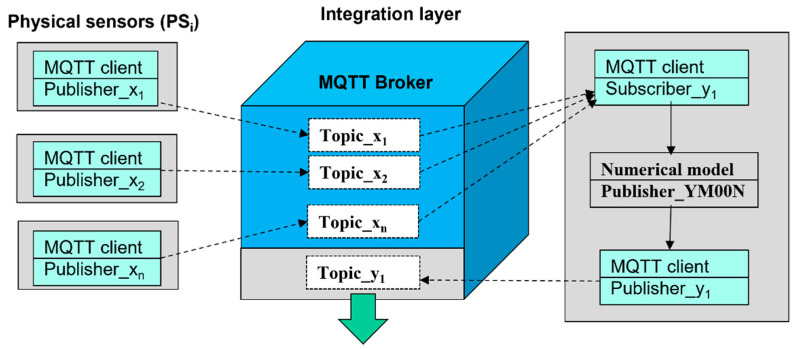
The concept of experimental workplace for simulation of virtual redundant sensors.

In addition to the subscriber role, the virtual redundant sensor has to have another connection to the broker, in the role of Publisher, so that it can publish its result to MQTT broker. The new value is written to the associated Topic_y of virtual redundant sensor (Figure 8). The control applications can access values of virtual redundant sensors in the same way as values of physical ones, via MQTT broker and associated Topic, in order to abstract implementation and virtual nature of the new sensor (digital twin to physical sensors).

As MQTT broker we chose Rabbit MQ software [27,28], which is mainly used as AMQP (Advanced Message Queuing Protocol) broker to support advanced messaging and queue management with high availability and performance. However, it supports additional protocols (MQTT) and features in the form of additional “plug-ins”.

To implement overall experimental solution, the following parts were used:

- ASP.NET script for emulated physical, virtual redundant sensors;
- Rabbit MQ as MQTT broker, as described before;
- MQTT clients for producers, consumers.

**Figure 9:**
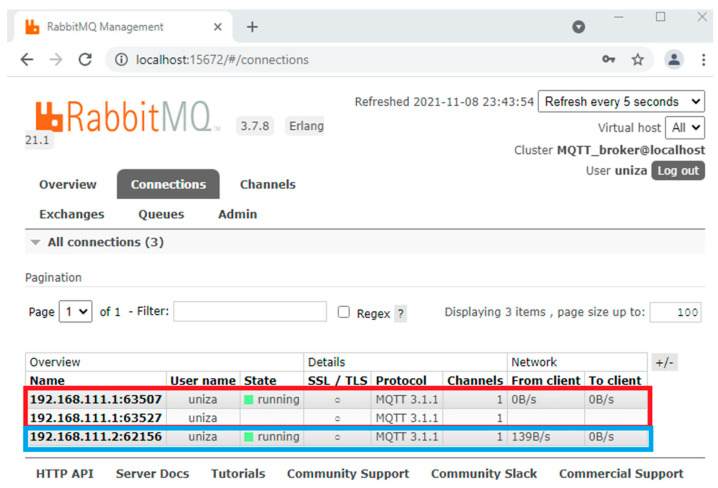
RabbitMQ as MQTT broker with sensor connections.

RabbitMQ (software management tool Rabbit MQ) management console is shown in Figure 9, with configured connections of the physical sensors (blue box, IP address: *192.168.111.2*…n) in publisher roles and the virtual redundant sensor in both publisher and consumer roles (*192.168.111.1*).

**Figure 10:**
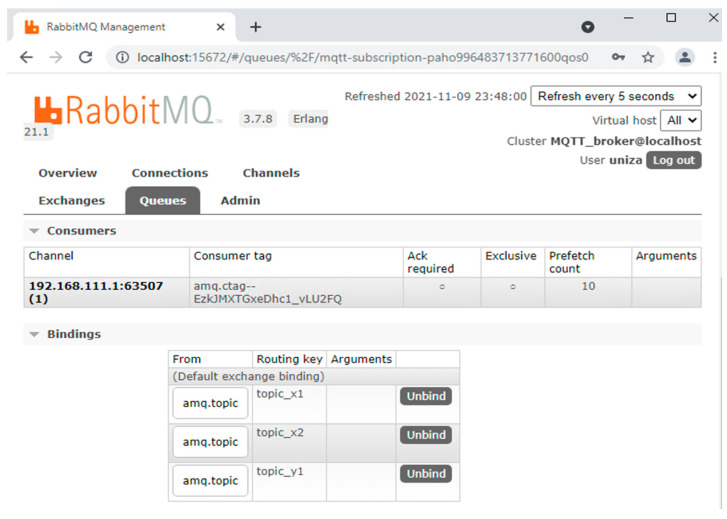
RabbitMQ queue setting for virtual redundant sensor as a consumer.

The consumer role requires proper setting of the AMQP (Advanced Message Queuing Protocol) queue (*amq.topic*) with routing keys that are mapped to MQTT topic of the sensors (topic_*x*1, topic_*x*2,…topic_*x_n_*) for IP address *192.168.111.1* of virtual redundant sensor, as shown in Figure 10.

In our experimental case, the physical sensors are emulated purely by software client, which can send manual input, or simulated values to MQTT broker via MQTT client in Producer role. A simulation of the input is based on the random value generator, which calculates deviation level for initial value, that is configurable on the screen of the emulated physical sensor (GUI). 

**Figure 11:**
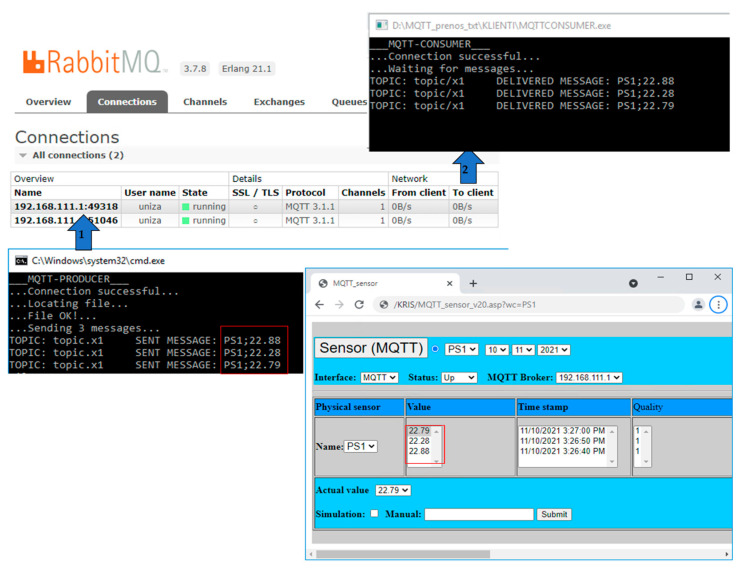
Emulated physical sensor with MQTT client.

Our implemented experimental workplace is shown in Figure 11. The emulated physical sensors generate measured values every 10 s to MQTT client, which sends them to MQTT broker with associated routing key “topic_*x*1”, that is assigned to MQTT queue (*amg.topic*) on the broker side and represents associated topic of the physical sensor PS1. The virtual redundant sensor is connected to MQTT broker via MQTT client in consumer role, reading values of connected physical sensors.

The simulations of proposed virtual redundant sensor were performed on the implemented experimental workplaces. The goal was to evaluate all numerical models (*m*oo*n*) with the emulated input from three physical sensors PS1–PS3. All sensors were represented by MQTT clients which were producing emulated measured values with updating of values as soon as the value has been changed in contrast to previous value. 

**Figure 12:**
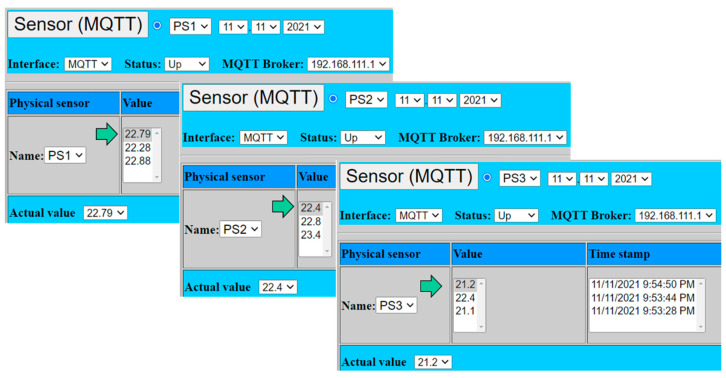
Emulated physical sensors connected to virtual sensor.

Figure 12 shows GUI of all three emulated sensors with the actual emulated values, which were sent to MQTT broker from emulated physical sensors as was illustrated by Figure 11.

- PS1: *x*_1_ = 22.79;
- PS2: *x*_2_ = 22.40;
- PS3: *x*_1_ = 21.20.

**Figure 13:**
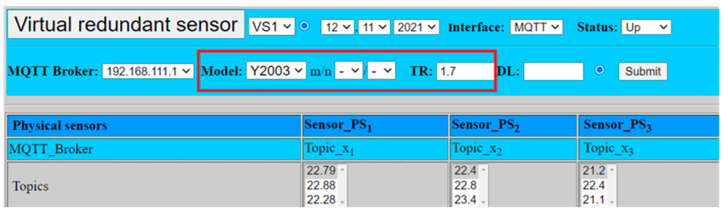
Emulated physical sensors connected to virtual sensor.

The most essential part of our work was dedicated to the creation of virtual redundant sensor software with support of defined numerical models in Equations (1) to (14). In order to enable a flexible simulation, the software client of the virtual redundant sensor supports selection of defined models with its required parameters. Virtual redundant sensor’s user interface (GUI—Graphical User Interface) is displayed in Figure 13.

GUI shows all actual values of connected physical sensors (*PS_i_*), which are mapped to associated topics on MQTT broker (Topic_*x_i_*), including the history of the last recent values (2 values back). The selection of required numerical models (*m*oo*n*) including the required parameters is marked by red box. 

For confirmation of the selected model, the submit button has to be activated. Software will automatically pre-select the desired model, confirming the selection of the model and its parameters in informational part of GUI: YM00N, *TR*. 

There is a need to select the following parameters:

- *n*—number of physical sensors (*n* ≥ 1 and *n* ≤ 3);
- *m*—number of evaluated sensors (*m* ≤ *n*, *n* ≥ 1 and *n* ≤ 3);
- *TR*—tolerance range (float number *x.y*);

As soon as the model is selected, in our case Y2003, software calculates the result value of the virtual sensor VSi. The calculated value is automatically transferred into associated topic on the MQTT broker (Topic_*y*_1_), which is to be consumed by fail-safe applications requiring the value’s validation. In addition, GUI provides quality status (validity) and time stamp so that evaluation of the results can be performed properly. In this most complex case, the values of all three sensors are to be evaluated and included into the overall calculated result. 

**Figure 14:**
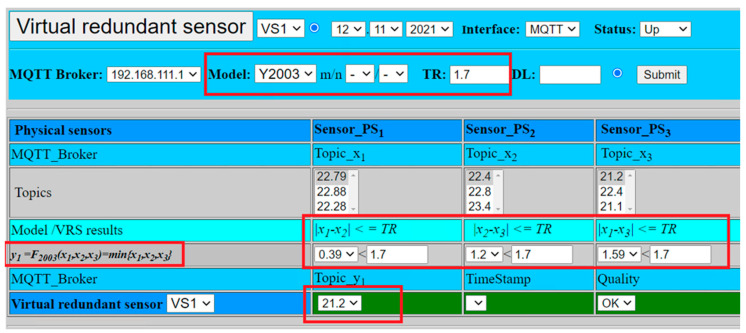
Virtual redundant sensor with fusion function *F*_2oo3_ and positive result.

Figure 14 illustrates one of the possible scenarios, when parameter *TR* = 1.7 and tolerable differences between all three sensors are met (0.39, 1.2, 1.59). Therefore, validation result is positive and can be set to OK. The result value is calculated as a minimum value out of all three sensor values (*VS*1 = 21.2), see equitation (22). This model would support positive validation result even in case of higher deviation from one of the sensors, the calculated result would be based on values from the remaining two sensors that met TR level, setting result value to the minimum of two sensors. The negative validation result would be calculated only if two sensors show deviation higher than the pre-defined *TR* value.

(22) $$VS1=y_{2\mathrm{oo}3}=F_{2\mathrm{oo}3}\left(x_{1},\;x_{2},\;x_{3}\right)=min\left\{22.79,\;22.40,\;21.20\right\}=21.20$$

In order to demonstrate the negative validation result, parameter TR was set to 1.0. In this case, the values from sensors PS1, PS2 show smaller deviation (0.39) than defined limit TR (1.0), however the differences $k_{2}$, $k_{3}$ between the values of the sensors PS2, PS3 (1.2) and PS1, PS3 (1.59) exceed the defined limit (1.0). Therefore, according to (7), (9), and (11), defined conditions are not met and the overall result has to lead to the negative validation (NOK). The value of the virtual sensor is set to zero (*VS*1 = 0), as shown by Figure 14 and the virtual sensor cannot provide the validated result.

(23) $$VS1=y_{2\mathrm{oo}3}=F_{2\mathrm{oo}3}\left(x_{1},\;x_{2},\;x_{3}\right)=0.$$

**Figure 15:**
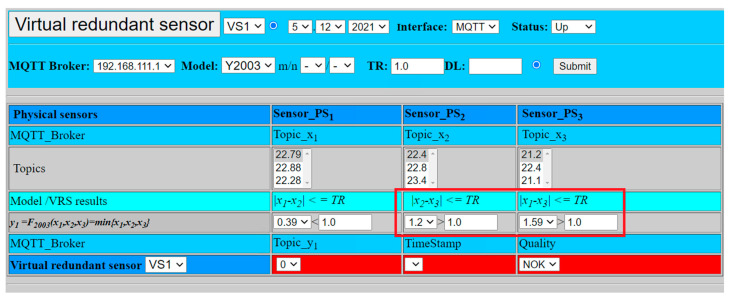
Virtual sensor with fusion function *F*_2oo3_ and negative result.

The experimental validation of our model was also extended for digital sensors with the logic signal. Figure 15 shows the simulation of the digital sensors with logic values:

- PS1: *x*_1_ = 1;
- PS2: *x*_2_ = 0;
- PS3: *x*_3_ = 1.

The result is calculated according to Formula (14):

(24) $$y_{2\mathrm{oo}3}=F_{2\mathrm{oo}3}\left(x_{1},\;x_{2},\;x_{3}\right)=1.$$

**Figure 16:**
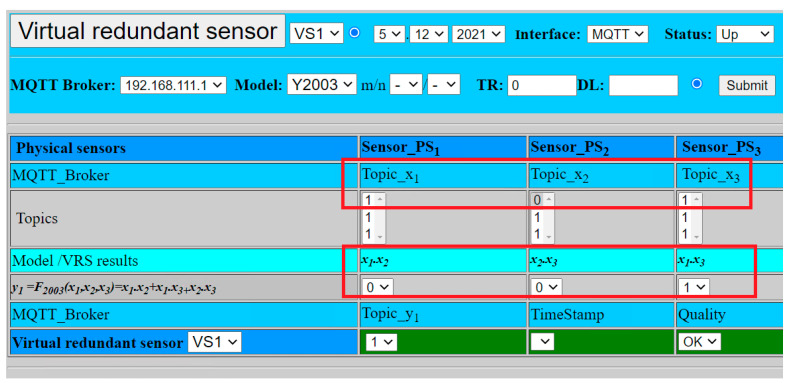
Virtual sensor with digital sensors and fusion function *F*_2oo3_.

In addition, the fusion function *F*_2oo3_ with voting gives the same result VS1 = 1, because two from three sensors provide the same signal log. 1, as shown in Figure 16.

The extended model of virtual redundant sensor was also experimentally verified regarding its redundancy and high-availability features. The second virtual sensor VS12, with open MQTT connections to all physical sensors and application, was activated in addition to VS11. The virtual sensor model with duplexing was selected for an experimental verification, due to necessity to deal with safety-related applications requiring very low $T_{ST}$ (Figure 7). The experiment was limited just to fusion function Y2002 with the aim to explore redundancy with duplexing of two virtual sensors VS11, VS12, using the same values from physical sensors as in previous experiments. 

**Figure 17:**
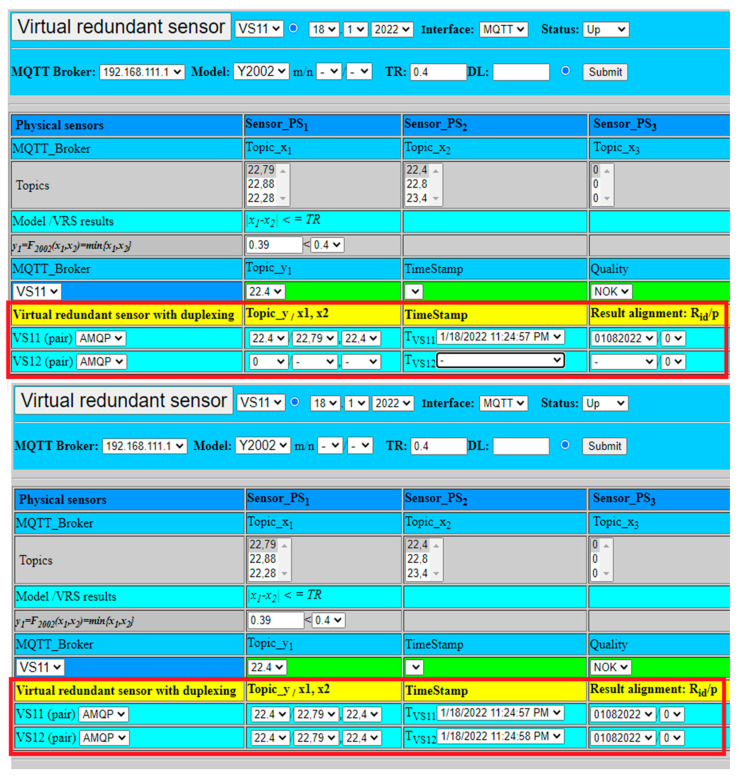
Virtual redundant sensor VS11 with duplexing with example of result confirmation.

VS11 virtual sensor validated result from two physical sensors. The selected fusion function provided as a result the minimum from physical sensors values (22.4), as shown in Figure 17. The result was sent via AMQP to VS12 virtual sensor for result alignment (25), which was checked by VS12 and confirmed (26). After confirmation, both results were published for application by two MQTT brokers.

(25) $$y_{1}=\left\{R_{id},\;T_{VS11},\;p\right\}=\left\{22.4,12.1.2022\;11:53:13\;PM,\;01082022,0\right\},$$

(26) $$y_{2}=\left\{R_{id},\;T_{VS12},\;p\right\}=\left\{22.4,12.1.2022\;11:53:14\;PM,\;01082022,0\right\}.$$

**Figure 18:**
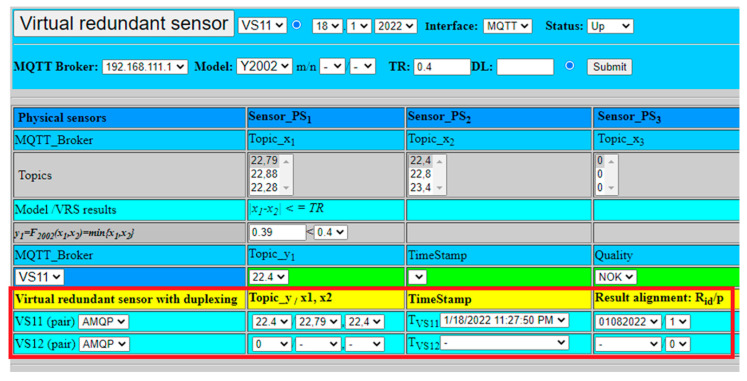
Virtual redundant sensor VS11 with duplexing and example of VS12 failure.

To check proper handling, the failure of VS12 virtual sensor has been simulated as well. In that case, VS11 did not get confirmation from VS12. VS11 consequently set parameter p=1 and published the result value to MQTT broker (27). The setting of p=1 notificated application to use exclusively just VS11 values and ignore VS12 values, till recovery with parameter p=0 is available again, as shown Figure 18.

(27) $$y_{1}=\left\{R_{id},\;T_{VS11},\;p\right\}=\left\{22.4,\;12.1.2022\;11:53:13\;PM,\;01082022,1\right\}.$$

## 6. Conclusions

In this article, we share our research results concerning the virtual redundant sensors, which are intended for SRCSs. The main idea was to extend and enrich the capabilities of physical sensors by additional information with focus on so-called RAMS parameters. Virtual redundant sensors with different safety and reliability properties can be created from a set of physical sensors. Depending on the specification of the requirements for RAMS parameters of specific application, the appropriate combination of physical sensors and the mathematical model can be selected. Our focus was paid on the creation and validation of the appropriate numerical models which would define fusion functions for those parameters. Having combined physical sensors values, we are able to apply various models to influence overall results and contribute to RAMS parameters. In addition, our model was extended in order to ensure the required redundancy of virtual sensors and provide high-availability solution to safety-related applications. Two approaches based on mirroring and duplexing of virtual sensors were elaborated.

In order to validate our proposed approach, we have chosen the simpler models (*m*oo*n*) with fusion functions $F_{2\mathrm{oo}2}$ and $F_{2\mathrm{oo}3}$. The virtual redundant sensors provided enriched value, which is calculated as the minimum (based on the requested safety level) or the mean value (based on the requested availability) from physical sensors’ values. This approach helped us to simulate the needed capabilities of virtual redundant sensors even with changing values in the defined time frame. Experimental evaluation was based on emulation of physical sensors connected to MQTT broker (Rabbit MQ), which was connected with virtual sensor instance in both roles of MQTT subscriber and publisher. The physical and virtual sensors had WEB-based graphical user interface (ASP.NET) to select the needed numerical model and its parameters (TR) to validate results within the defined tolerance range or decision level. Our implemented experimental solution supports both implementation scenarios (Cloud based, Edge based). The experimental solution utilized AMQP protocol for the extended model with duplexing of virtual sensors, result alignment ($R_{id}$) and verification (p). The used products (MQTT broker) and platforms can be chosen according to specific use-cases and required parameters. 

The results of testing have shown that the proposed virtual redundant sensor concept can be applied to improve RAMS parameters of physical sensor. Our fusion functions calculated results according to selected model in all cases. The proposed model can handle also failure of one or more physical sensors. Not standard cases were marked with quality status “Not OK” and result value was set according to model to 0, as documented by attached GUI screens of the virtual redundant sensor. The extended model with duplexing was fully verified and embedded redundancy enabled virtual sensor operation without interruption of delivery validated values when one of the virtual sensors failed, so overall solution confirmed requested virtual redundant sensor capabilities.

All in all, the proposed concept of the virtual redundant sensor can provide additional value to safety-relevant systems, simplifying control application by validating sensors results and providing higher level of safety level and availability for safety related processes. However, this conceptual work just justifies the proposal itself. For real applications, we would have to consider a more complex definition of the fusion functions, because our used minimum/average values can be used for specific processes, but do not represent generally all processes (different logic required by process). Therefore, our future work could be oriented to incorporate flexible creation of fusion functions in form of library, or via “plugin” concept. As well as there is a potential to extend the model for dynamic changes of sensor values, that have not been simulated in our experiment. With respect to extended model, the duplexing approach requires physical sensors and application to have dual MQTT connection to both virtual sensors. This precondition might not be possible in all use-cases. Therefore, mirroring concept and its experimental verification shall be elaborated as well in future work, mainly if $T_{ST}$ parameter can be accepted by safety-related application.
